# *Saussurea involucrata* (Snow Lotus) *ICE1* and *ICE2* Orthologues Involved in Regulating Cold Stress Tolerance in Transgenic *Arabidopsis*

**DOI:** 10.3390/ijms221910850

**Published:** 2021-10-07

**Authors:** Chia-Ling Wu, Lee-Fong Lin, Hsiao-Chun Hsu, Li-Fen Huang, Chung-Der Hsiao, Ming-Lun Chou

**Affiliations:** 1 Department of Life Sciences, Tzu Chi University, Hualien 97004, Taiwan; 106726102@gms.tcu.edu.tw (C.-L.W.); leelin@gms.tcu.edu.tw (L.-F.L.); 2 The Society of Wilderness, Taipei 10073, Taiwan; debby@wilderness.tw; 3 Graduate School of Biotechnology and Bioengineering, Yuan Ze University, Zhongli, Taoyuan 32003, Taiwan; hlf326@saturn.yzu.edu.tw; 4 Department of Bioscience Technology, Chung Yuan Christian University, Chung-Li 320314, Taiwan; cdhsiao@cycu.edu.tw

**Keywords:** ICE orthologues, cold stress tolerance, *CBF* expression, snow lotus

## Abstract

As with other environmental stresses, cold stress limits plant growth, geographical distribution, and agricultural productivity. *CBF/DREB* (*CRT*-binding factors/*DRE*-binding proteins) regulate tolerance to cold/freezing stress across plant species. ICE (inducer of *CBF* expression) is regarded as the upstream inducer of *CBF* expression and plays a crucial role as a main regulator of cold acclimation. Snow lotus (*Saussurea involucrata*) is a well-known traditional Chinese herb. This herb is known to have greater tolerance to cold/freezing stress compared to other plants. According to transcriptome datasets, two putative *ICE* homologous genes, *SiICE1* and *SiICE2*, were identified in snow lotus. The predicted *SiICE1* cDNA contains an ORF of 1506 bp, encoding a protein of 501 amino acids, whereas *SiICE2* cDNA has an ORF of 1482 bp, coding for a protein of 493 amino acids. Sequence alignment and structure analysis show SiICE1 and SiICE2 possess a S-rich motif at the N-terminal region, while the conserved ZIP-bHLH domain and ACT domain are at the C-terminus. Both *SiICE1* and *SiICE2* transcripts were cold-inducible. Subcellular localization and yeast one-hybrid assays revealed that SiICE1 and SiICE2 are transcriptional regulators. Overexpression of *SiICE1* (*35S::SiICE1*) and *SiICE2* (*35S::SiICE2*) in transgenic *Arabidopsis* increased the cold tolerance. In addition, the expression patterns of downstream stress-related genes, *CBF1*, *CBF2*, *CBF3*, *COR15A*, *COR47*, and *KIN1*, were up-regulated when compared to the wild type. These results thus provide evidence that SiICE1 and SiICE2 function in cold acclimation and this cold/freezing tolerance may be regulated through a CBF-controlling pathway.

## 1. Introduction

In contrast to animals, plants are not able to move to a suitable environment in order to survive. When facing a vital change in environment and climate, such as different stresses, plants might try to adapt to and survive in such an environment. Biotic stresses include infection of insects, fungi, and pathogens, while major abiotic stresses are light strength, temperature, drought, salt concentration, and hypoxia. Among these, the optimal growth temperature is between 0~45 °C for most plant species on earth [1,2]. Cold/freezing stress not only restricts plant growth and geographical distribution, but also causes major effects on agricultural production and crop quality [3,4,5]. Low temperatures result in plant cell dehydration and tissue damage, thereby causing the denaturation of cellular proteins. In addition, cold stress contributes to increased cellular permeability, which in turn gives rise to an abnormal metabolism, leading to plant death [6,7]. Chloroplast is the first cold sensitive organelle in a plant cell. Cold stress causes a change in both the composition and structure of the photosystems in the thylakoid membrane, leading to obstructed light reactions of photosynthesis. Once photosynthesis is hindered, plant cell development is highly affected [8]. In addition to causing delayed cellular development, cold stress results in plants flowering early, flower abscission, and fruit drop shedding, all of which are harmful for the reproduction of flowering plants [9,10,11].

Cold stress is classified as chilling (0–15 °C) and freezing (<0 °C) stresses. Plants can increase cold tolerance by exposure to chilling or non-freezing temperatures in temperate regions by cold acclimation. Generally, plants originating from temperate regions, such as *Arabidopsis*, elicit a changeable degree of cold tolerance and can gradually increase their freezing tolerance with time during exposure to cold and non-freezing temperatures. This process is known as cold acclimation [12]. In contrast to this, plants of tropical and subtropical origins are sensitive to cold stress and lack the cold acclimation mechanism. Earlier studies have shown that several genes can be activated in response to cold/freezing stress [12,13,14]. For example, *kinI* gene expression was up-regulated up to 20× in response to cold treatment in *Arabidopsis*. *COR*s (cold-regulated or cold-responsive genes) were deduced to be the regulator genes responsible for cold tolerance [15,16,17]. *COR15A*, *COR15B*, and *COR47* were identified to be cold-related genes. These genes were further proved to have the capacity to increase plant tolerance to cold/freezing stress [18,19,20]. It is of interest to note that the gene expression of *RD29A/COR78* is up-regulated in drought, low-temperature, or high-salt conditions. On the contrary, *RD29B* gene expression is not induced by cold stress, and its expression is up-regulated only after a long duration (10 h) of high-salt treatment [21]. Plant hormone abscisic acid (ABA) was known to be related to cold acclimation. The promoter region of *RD29A/COR78* contains two DRE (dehydration-responsive) cis-elements and one ABA-responsive element (ABRE), whereas *RD29B* only contains the presence of an ABRE. Thus, the gene expression of *RD29A/COR78* is controlled by the C-repeat (CRT)/dehydration-responsive element (DRE) (CRT/DRE) cis-element which interacts with the upstream transcription factor, causing gene activation in response to cold, dehydration, or high-salt stress [21]. However, expression of *RD29B* is induced through ABA signaling pathway under a dehydration or high-salt environment [22]. Following analysis, the promoter region of cold- and dehydration-related genes contains one or more CRT/DRE cis-elements with a core sequence of CCGAC, such as that of *COR15* and *RD29A/COR78* genes [21,23,24]. After the binding of a transcription factor to a CRT/DRE cis-element, a downstream gene is activated and results in increased tolerance to cold and drought stress. Two types of transcription factors are classified; one is named C-repeat binding factor/DRE-binding protein 1 (CBF/DREB1) which is induced by cold stress, but not by drought or high-salt stress [25]. On the contrary, the other is called DRE-binding protein 2 (DREB2), which is increased under drought or high-salt conditions [26].

Several lines of evidence have shown that a cold signaling network increases cold tolerance. Thus, it has been suggested that the ICE1-CBF-COR regulatory cascade is one of the most critical signaling pathways in *Arabidopsis* [12,27,28]. According to Gilmour and his coworkers’ studies [29], *CBF* mRNA transcripts start to be accumulated after cold treatment for 15 min. An inducer of CBF expression gene (*ICE*) was identified by using the *CBF3-Luc* platform and genetic screening [30,31]. A *ICE1*-defective gene results in reduced gene expression related to tolerance to cold and dehydration [31]. Furthermore, ICE1 is identified to be a positive regulator of *CBF* expression based on the data of an electrophoresis mobility shift assay (EMSA)/transient expression assay and the overexpression of *ICE1*, which enhances freezing tolerance in transgenic plants [31]. Moreover, microarray data exhibit 939 genes related to cold regulation. Among these, 655 genes are up-regulated, while 284 genes are down-regulated. These early cold-responsive gene products are mostly transcription factors which are involved in the activation of subsequent late cold-responsive genes [32]. *ICE1* in *Arabidopsis* is named *AtICE1* according to TAIR (The Arabidopsis Information Resource) website with the locus of AT3G26744. It is located at the third chromosome with a gene product of 494 amino acids, which contains a highly conserved basic helix–loop–helix (bHLH) domain, belonging to the MYC-type transcription factor family [33]. Two different functional motifs are located within the bHLH domain. Basic motif sites are located at the N-terminal end with 15 highly conserved amino acids, which are implicated in the DNA–protein interaction. The other HLH motif is located at the C-end, which is rich in hydrophobic amino acids, forming two amphipathic alpha-helices connected by a loop. This motif is involved in protein dimerization and the following binding to DNA [34]. Homologous *AtICE2* gene (AT1G12860), located at the first chromosome, was found in *Arabidopsis* based on the protein sequence similarity. This gene product is composed of 450 amino acids, and contains a similar bHLH domain [33,35]. Overexpression of *AtICE2* (superHALF2-ICE2) transgenic *Arabidopsis* exhibits increased tolerance to cold/freezing treatment (4 °C acclimation, following −20 °C stress) when compared to the wild type. In addition, overexpressed AtICE2 causes the increased expression of the downstream *CBF1* gene, indicating that AtICE2 is involved in cold tolerance through controlling *CBF* gene expression under cold stress [35]. Most recently, *AtICE* homologs have subsequently been found in several other plant species, and these ICE-like proteins, when overexpressed in transgenic plants, were revealed to increase stress tolerance [36,37,38,39,40,41,42,43,44,45]. For example, dehydrin-like gene (*SiDhn2*) and stearoyl-acyl carrier protein desaturase (*SikSACPD*), related to cold acclimation in snow lotus (*S. involucrata*), have been cloned, and transgenic plants showed greater resistance to freezing and drought stress than the wild-type plants [46,47].

Snow lotus (*S. involucrata*) is a well-known traditional Chinese medicinal herb which possesses an enhanced plant tolerance to cold/freezing stress in its native habitat, leading to its capability to grow in high mountain areas covered with snow throughout the year. Advances in next-generation sequencing (NGS) technologies and the sequencing data obtained are useful to investigate the genome-wide and transcription expression profile of non-model species such as *S. involucrata* [48,49]. Based on re-assembled snow lotus transcriptome datasets, we identified two putative *ICE* homologous genes, *SiICE1* and *SiICE2*, which were revealed to be phylogenetically similar to other plant *ICE* genes. Herein, we isolated these two genes and functionally characterized their roles in cold tolerance by overexpressing them in *35S::SiICE1* (L7 and L14) and *35S::SiICE2* (L10 and L17) transgenic *Arabidopsis*. Our studies revealed that SiICE1 and SiICE2 of snow lotus play an important role as positive regulators in cold acclimation.

## 2. Results

### 2.1. Structural Analysis of ICE Proteins from Snow Lotus and Arabidopsis

**Figure 1:**
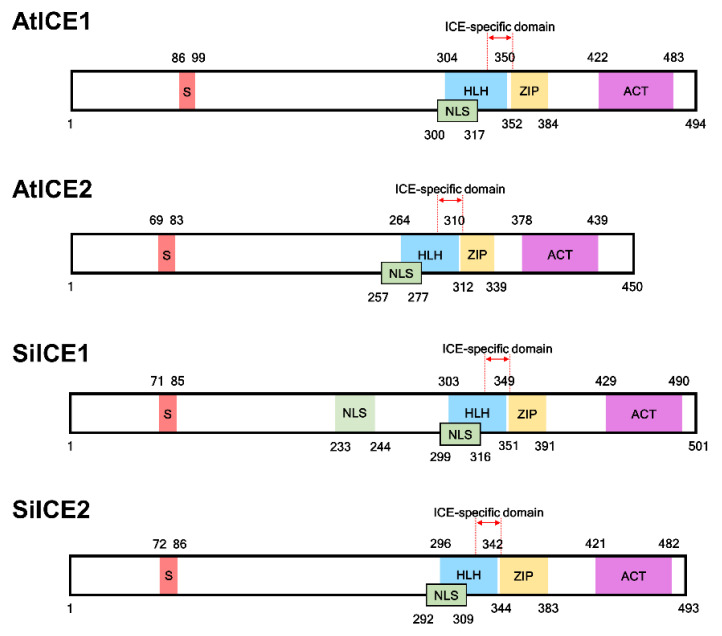
Structural comparison among SiICE1, SiICE2, AtCE1, and AtICE2 in snow lotus and *Arabidopsis*, respectively. Red region indicates serine (S)-rich area. Blue area is helix–loop–helix (HLH). Yellow box represents zipper region (ZIP). Purple shows the ACT_UUR_ACR-like (ACT) region. Numbers located on the top/bottom of the boxes with different colors are the numbers of amino acids within each ICE protein. NLS: nuclear localization signal.

**Figure 2:**
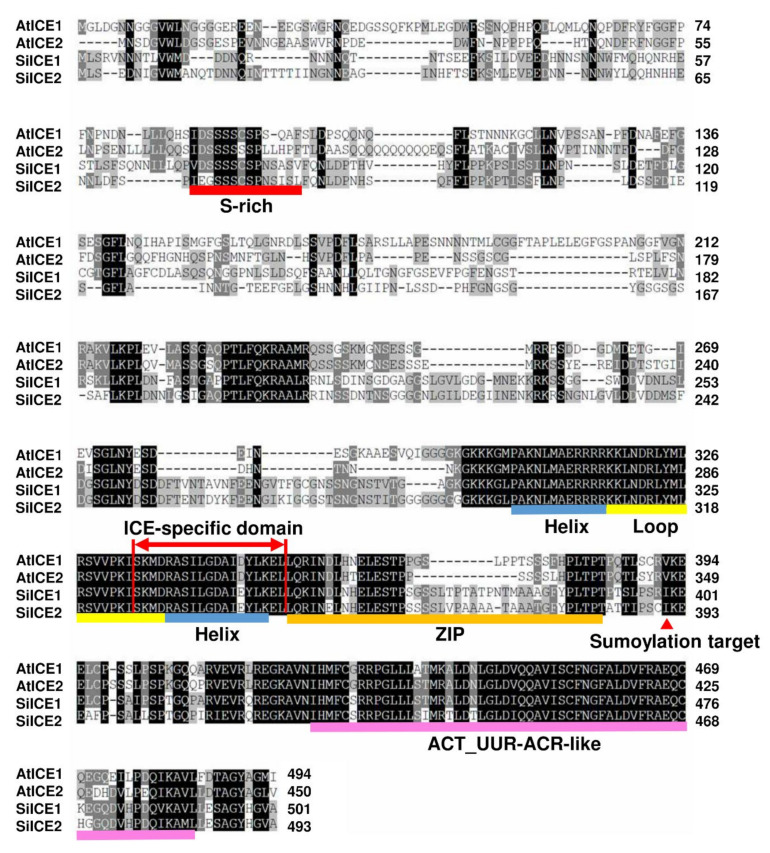
Amino acid sequence alignment and analysis of SiICE1, SiICE2, AtICE1, and AtICE2 by employing ClustalW2 program. Black area indicates regions with amino acid identity among these ICE proteins. Gray box exhibits where physically and chemically similar amino acids are replaced within different ICE proteins. Different colors are used to underline the distinct predicted structures. Red line shows serine-rich (S-rich) region; blue–yellow–blue line indicates helix–loop–helix (HLH) domain; orange is zipper region (ZIP domain); and light-purple represents ACT_UUR_ACR-like (ACT) structure. ICE-specific domain is shown as double red arrows. Red arrowhead symbolizes the target site (lysine) for sumoylation.

The gene expression profile of snow lotus transcriptome was re-constructed according to the methods described in the Materials and Methods section. Thereafter, the re-constructed gene sequences were used as the searching database in order to investigate the potential gene candidates corresponding to cold acclimation and cold tolerance. SiICE1 and SiICE2 (Saussurea involucrata inducer of CBF expression 1 and 2), genes homologous to AtICE1 and AtICE2, respectively, were identified by using this re-constructed transcriptome dataset of snow lotus. The full-length cDNA of SiICE1 contains 1506 bps, encoding a protein of 501 amino acids with the molecular weight (*M*_W_) of 54.3 kDa and a pI of 5.81, whereas cDNA of SiICE2 is composed of 1482 bps, coding for a protein of 493 amino acids, *M*_W_ of 53.2 kDa, and a pI of 5.28. The complete gene sequences of AtICE1 and AtICE2 of *Arabidopsis* were taken from The Arabidopsis Information Resource (TAIR) with the gene loci of AT3G26744 [31,32] and AT1G12860 [33], respectively. Structural analysis of different ICE proteins, including SiICE1 and SiICE2, exhibited similar functional domains, such as serine-rich (S-rich) region sites at the N-terminal end, while the bHLH-ZIP domain, ICE-specific domain, and ACT_UUT-ACR (ACT) domains were located at the C-terminus (Figure 1 and Figure 2). A nuclear localization signal (NLS) was located for SiICE1 and SiICE2, respectively, by using the website of the Machine Learning and Evolution Laboratory (http://mleg.cse.sc.edu/seqNLS/ (accessed on 6 January 2021)). The NLSs were predicted to be between 233–244 and 299–316 amino acids, respectively, for SiICE1, while between 292–309 amino acids for SiICE2 (Figure 1).

**Figure 3:**
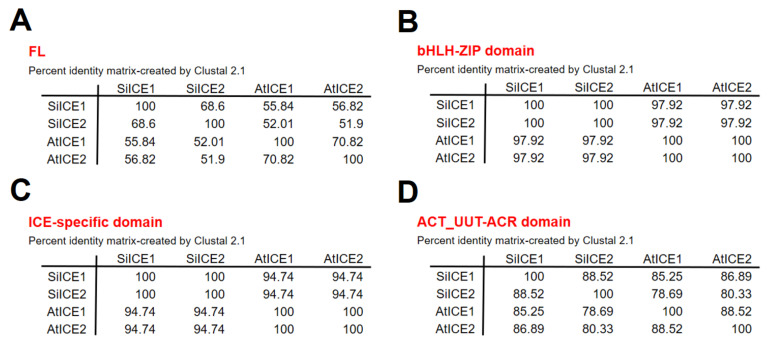
Identity comparison of amino acid sequences of different ICE proteins, including AtICE1, AtICE2, SiICE1, and SiICE2. (**A**) Comparison of full-length (FL) amino acid sequences of four different ICE proteins. (**B**) Comparison of the bHLH-ZIP domain. (**C**) Comparison of the ICE-specific domain. (**D**) Comparison of the ACT_UUT-ACR (ACT) domain.

**Figure 4:**
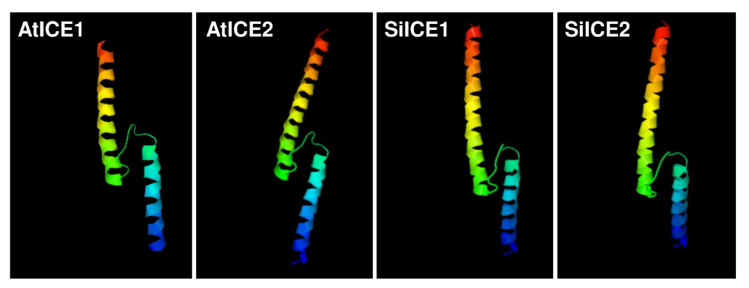
Simulated three-dimensional structure of a bHLH-ZIP dimer located within AtICE1, AtICE2, SiICE1, and SiICE2 proteins, respectively, obtained by using the VMD program. Red/yellow/green and green/blue indicate a helix structure, respectively.

Figure 3 shows the amino acid sequence blast results of these homologous ICE proteins. The total identity shared between SiICE1 and SiICE2 is 68.6%, while that between SiICE1 and AtICE1 or AtICE2 is 55.84% and 56.82%, respectively. The similarity of SiICE2 and AtICE1 or AtICE2 is 52.01% and 51.90%, respectively. Figure 3B,C elicit much higher degrees of shared identity for the bHLH-ZIP domain and ICE-specific domain, with 97.92% and 94.74% being the same, respectively, within ICE proteins of different species. Finally, the similarity of the ACT_UUT-ACR (ACT) domain of SiICE1 to that of AtICE1 or AtICE2 shows 85.25% and 86.89% shared identity, while SiICE2 compared to AtICE1 or AtICE2 is 78.69% and 80.33% shared identity, respectively. SiICE1 and SiICE2 contain 88.52% shared identity within the ACT domain. In addition to the highly shared amino acid sequence identity of the bHLH-ZIP domain among four different ICE proteins (Figure 3B), their simulated three-dimensional structures show high similarity within this domain when the Visual Molecular Dynamics (VMD) program was used (Figure 4).

### 2.2. Phylogenetic Tree of SiICE1, SiICE2, and Other ICE Proteins

**Figure 5:**
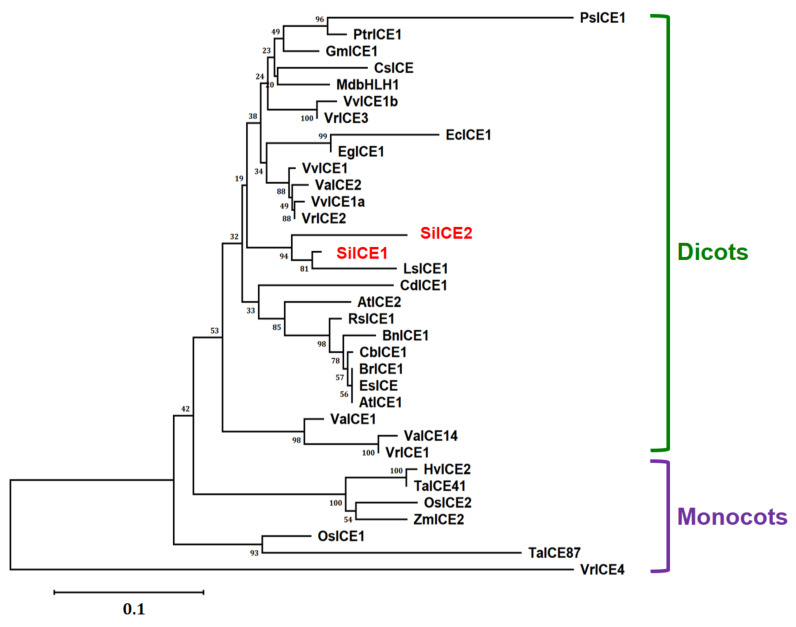
Phylogenetic analysis of SiICE1 and SiICE2 with orthologous ICE proteins from other plant species, including dicots and monocots. The full-length amino acid sequence alignment of SiICE1, SiICE2, and other ICE proteins was analyzed by using the ClustalW2 program, followed by MEGA X counted 1000 times using the neighbor-joining method [50,51,52]. A phylogenetic tree was then constructed. Except for SiICE1 and SiICE2 of *Saussurea involucrata*, others include AtICE1 (NP_189309) and AtICE2 (NP_172746) of *Arabidopsis thaliana*, BnICE1 (AEL33687) of *Brassica napus*, BrICE1 (ACB70963) of *Brassica rapa*, CbICE1 (AAS79350) of *Capsella bursa-pastoris*, CsICE (ACT90640) of *Camellia sinensis*, EcICE1 (ADY68776) of *Eucalyptus camaldulensis*, EgICE1 (AEF33833) of *Eucalyptus globulus*, EsICE (ACT68317) of *Eutrema salsugineum*, GmICE1 (ACJ39211) of *Glycine max*, HvICE2 (ABA25896) of *Hordeum vulgare*, LsICE1 (ADX86750.1) of *Lactuca sativa*, MdbHLH1 (ABS50251) of *Malus domestica*, OsICE1(Os11g0523700) and OsICE2 (Os01g0928000) of *Oryza sativa*, PsICE1 (ABF48720) of *Populus suaveolens*, PtrICE1 (ABN58427) of *Populus trichocarpa*, RsICE1 (ADY68771) of *Raphanus sativus*, TaICE41 (ACB69501) and TaICE87 (ACB69502) of *Triticum aestivum*, VaICE1 (AGP04217), VaICE2 (AGP04218), and VaICE14 (ADY17816) of *Vitis amurensis*, VvICE1 (AFI49627), VvICE1a (AGQ03810), and VvICE1b (AGQ03811) of *Vitis vinifera*, VrICE1 (AGG34704), VrICE2 (AIA58705), VrICE3 (AIA58706), and VrICE4 (AIA58707) of *Vitis riparia*, and ZmICE2 (ACG46593) of *Zea mays*.

Different homologous ICE genes were identified from distinct plant species based on the NCBI gene/protein database. Full-length amino acid sequences of SiICE1 and SiICE2 from snow lotus and those of other ICE proteins were analyzed for their phylogenetic relationship by using ClustalW multiple sequence alignment, followed by being counted 1000× using the neighbor-joining method of Molecular Evolution Genetic Analysis (MEGA) X [50,51,52]. Thereby, a phylogenetic tree was obtained and shown in Figure 5. Two different groups were classified as dicots and monocots except for the VrICE4 protein (Figure 5). From the phylogenetic tree, SiICE1 and SiICE2 belong to the dicots and share high similarity with LsICE1, with a homology of 95% and 96%, respectively. In addition, SiICE1 and SiICE2 are relatively similar to AtICE1 and AtICE2 based on the phylogenetic tree, which is in agreement with the results of amino acid sequence alignment (Figure 3 and Figure 5).

### 2.3. SiICE1 and SiICE2 Differential Gene Expression in Response to Cold Stress in the Callus of Snow Lotus

**Figure 6:**
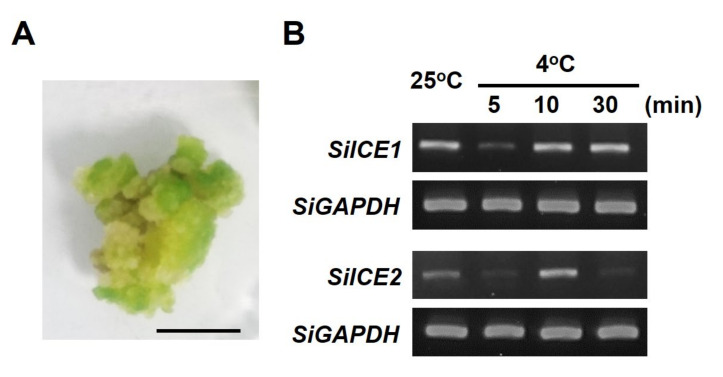
*SiICE1* and *SiICE2* differential gene expression in response to cold stress in the callus of snow lotus. Snow lotus callus cultivated for 2 weeks under long-day/light conditions, then dark treatment for 1 week, was divided into two groups. The control group was untreated and cultured at 25 °C, whereas the experimental group was low-temperature treated for 5, 10, or 30 min at 4 °C, respectively. Thereafter, mRNA expression of *SiICE1* and *SiICE2* was examined, respectively. (**A**) Phenotype of a snow lotus callus. Scale bar = 1 cm. (**B**) Semi-qPCR analysis of *SiICE1* and *SiICE2* gene expression. *SiGAPDH* represents the reference control of gene expression.

In order to explore the involvement of *SiICE1* and *SiICE2* genes in response to low-temperature stress, callus of snow lotus was cultivated and used as an experimental material when the live whole plant was not able to be obtained. (Figure 6A). Snow lotus callus was cultivated for 2 weeks under long-day/light conditions (16 h light/8 h dark), was then subjected to dark treatment for 1 week, and then divided into two groups. The control group was untreated and cultured at 25 °C, whereas the experimental group was low-temperature treated for 5, 10, or 30 min at 4 °C, respectively. A specific primer set was designed for semi-quantitative PCR (semi-qPCR), and *SiICE1* and *SiICE2* gene expression was determined in snow lotus callus. As shown in Figure 6B, both *SiICE1* and *SiICE2* genes were normally expressed at 25 °C. These PCR products were sequenced and identified to be the correct gene sequences of *SiICE1* and *SiICE2*, respectively. Following the moving of room temperature of cultivated callus from 25 °C to 4 °C for 5 min, both *SiICE1* and *SiICE2* gene expression was rapidly decreased, which increased again after 10 min of cold treatment. After 30 min of cold stress, *SiICE1* gene expression maintained a similar level. However, *SiICE2* gene expression was significantly reduced after 30 min treatment at 4 °C, even lower than that of the control group (25 °C) (Figure 6B). Nevertheless, cold stress results in differential gene expression of *SiICE1* and *SiICE2*, suggesting that these two genes may have a similar function to that of *AtICE1* and *AtICE2*, implicated in cold regulation.

### 2.4. Nuclear Localization of SiICE1 and SiICE2 Proteins

**Figure 7:**
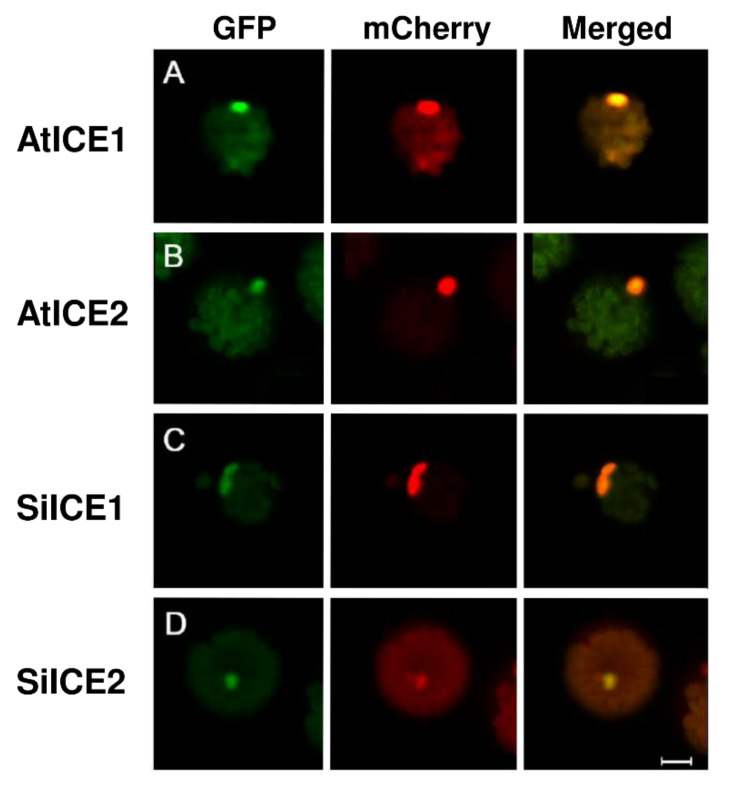
Nuclear localization of GFP-SiICE1 and GFP-SiICE2. A green fluorescent protein (GFP) gene was used as a reporter gene which is transiently expressed in the protoplast of *Arabidopsis*. The localization of GFP-SiICE1and GFP-SiICE2 fusion proteins is then shown under the confocal microscope by the presence of green fluorescence. Left panel: green fluorescence represents the location site of (**A**) AtICE1, (**B**) AtICE2, (**C**) SiICE1, and (**D**) SiICE2, respectively. Middle panel: mCherry with red fluorescence indicates the location of AtCO-mCherry, which is used as a positive control for the nuclear localization assay. Right panel: merged image indicates the superimposition of GFP and mCherry. The white proportional scale symbolizes 10 μm.

Earlier studies have revealed that AtICE1 and AtICE2 of *Arabidopsis*, and VaICE1 and VaICE2 of *Vitis amurensis* are the members of the same MYC-type bHLH transcription factor family, meaning that they can enter the nucleus and regulate the downstream gene expression, respectively [41]. According to Figure 1 of our study, a nuclear localization signal (NLS) is predicted to be present in both SiICE1 and SiICE2 of snow lotus. In order to examine if the NLS can truly lead these ICE proteins to the nucleus, we constructed recombinant plasmids carrying different fluorescent marker genes as the reporter genes, which are fused with the distinct ICE genes. Plasmids with a CaMV35S promoter followed by a green fluorescent protein (GFP) gene at the N-terminal end are fused with a SiICE1 or SiICE2 gene at the C-end, respectively. After translation, both GFP-SiICE1 and GFP-SiICE2 fusion proteins are produced. In a similar manner, GFP-AtICE1 and GFP-AtICE2 are constructed and made as well. An AtCO transcription factor was found that previously entered the nucleus, which plays an important role in improving flowering during photoperiodism in *Arabidopsis* [53,54]. Thus, a CONSTANS (AtCO) gene was fused to mCherry, which is a reporter gene, and its expression is regulated by the CaMV35S promoter. Thus, an AtCO-mCherry fusion protein with red fluorescence was used as a positive control in a nuclear localization assay. Figure 7 exhibits fluorescent ICE fusion proteins with a green (GFP-AtICE1, GFP-AtICE2, GFP-SiICE1, and GFP-SiICE2; A, B, C, and D) or red (AtCO-mCherry; middle panel) color under a confocal microscope. The right panel shows the merged results of GFP and mCherry, indicating that SiICE1 and SiICE2, as with AtICE1 and AtICE2, enter the nucleus and thus show one of the characteristic functions of transcription factors.

### 2.5. Transcription Activator Activity of SiICE1 and SiICE2

**Figure 8:**
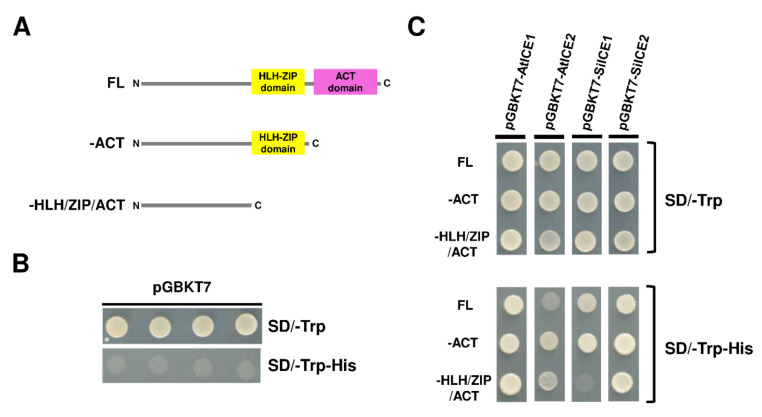
Yeast one-hybrid analysis of SiICE1, SiICE2, AtICE1, and AtICE2 with full-length (FL), –ACT, or –HLH-ZIP domain. (**A**) Orthologous *ICE* genes, including *SiICE1*, *SiICE2*, *AtICE1*, or *AtICE2*, were constructed in the pGBKT7 vector. Thus, different *ICE* genes carrying full-length (FL), lacking ACT (–ACT), or missing both ACT and HLH-ZIP (–HLH/ZIP/ACT) domains were inserted into the pGBKT7 vector, respectively. (**B**) Only the plasmid pGBKT7 vector was transformed into yeast strain AH109. Four different clones were selected and cultured in media SD/-Trp or SD/-Trp-His and used as a negative control. (**C**) *SiICE1*, *SiICE2*, *AtICE1*, or *AtICE2* genes carrying full-length (FL), –ACT, or –HLH/ZIP/ACT domains in pGBKT7 vector were transformed into AH109 yeast cells, respectively. Colony formation was then examined in the selection media SD/-Trp and SD/-Trp-His.

In the previous nuclear localization assay, SiICE1 and SiICE2 were revealed to be able to enter the nucleus and thus may function as transcription activators. In other words, SiICE1 and SiICE2 should contain an activation domain (AD) which activates downstream gene expression. In order to prove so, SiICE1 and SiICE2 genes were cloned into a pGBKT7 vector, respectively. The plasmid pGBKT7 vector itself not only contains a GAL4 DNA binding domain (DNA-BD) which is responsible for binding to its corresponding promoter region of the downstream controlling gene, but also carries a TRP1 nutritional marker for the selection of vector-transformed yeast cells. Thus, pGBKT7-AtICE1, pGBKT7-AtICE2, pGBKT7-SiICE1, and pGBKT7-SiICE2 with FL, –ACT, or –HLH/ZIP/ACT were constructed, respectively (Figure 8A). These respective recombinant DNA were then transformed into a yeast AH109 strain and cultured in agar plates without the amino acid tryptophan (SD/-Trp) or without both tryptophan and histidine (SD/-Trp-His) (Figure 8C). Vector pGBKT7 itself was transformed into yeast cells as a negative control. As shown in Figure 8B, yeast cells grew in the absence of the amino acid Trp (SD/-Trp), indicating vector pGBKT7 was successfully transformed into yeast cells and that the amino acid Trp is synthesized in these cells. However, the yeast only carried the vector and was not able to grow in the absence of both Trp and His (SD/-Trp-His), because the vector does not contain an activation domain (AD), which is required to activate the expression of the *HIS* reporter gene located within the yeast AH109 strain (Figure 8B). On the contrary, all the pGBKT7-AtICE1, pGBKT7-AtICE2, pGBKT7-SiICE1, and pGBKT7-SiICE2 with FL amino acids grew in SD/-Trp-His, suggesting that these ICE proteins carry an AD and function as transcription activators (Figure 8C). To further investigate the location of Ads within these ICE proteins, different deletion mutants were generated as shown in Figure 8A. A yeast one-hybrid assay locates the AD within the HLH-ZIP domain for pGBKT7-SiICE1, while within the N-terminal end (-HLH/ZIP/ACT) of pGBKT7-AtICE1, pGBKT7-AtICE2, and pGBKT7-SiICE2 (Figure 8C). Thus, this study revealed that AD sites are mainly located at the N-terminal end of different ICE proteins, except SiICE1 in which the AD is located within the bHLH-ZIP domain.

### 2.6. Overexpression of SiICE Genes in Transgenic Arabidopsis under Normal Growth Conditions or Cold Stress

**Figure 9:**
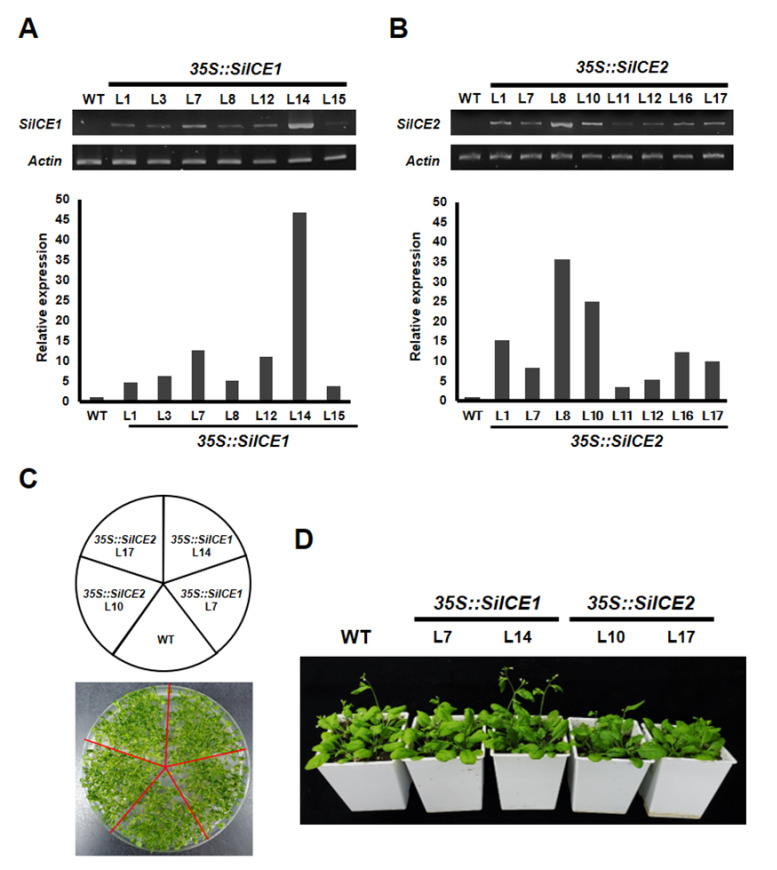
Semi-qRT-PCR of *SiICE1* and *SiICE2* expression in transgenic *Arabidopsis*. (**A**) Seven *35S::SiICE1* transgenic plants, L1, L3, L7, L8, L12, L14, and L15, were randomly selected and semi-qRT-PCR reactions were performed. Wild type (WT) was used as a control group, while *Actin2* was employed as a reference control for gene expression at the transcriptional level. (**B**) Similarly, a semi-qRT-PCR assay was conducted for *SiICE2* expression, including L1, L7, L8, L10, L11, L12, L16, and L17 *35S::SiICE2* transgenic plants. The lower panel shows the quantitative results of 9A and 9B, respectively. (**C**,**D**) Phenotypes of the WT, *35S::SiICE1* (L7, L14), and *35S::SiICE2* (L10, L17) transgenic plants. Seeds for different transgenic *Arabidopsis* were planted in 1/2 MS agar plates for 2 weeks (shown in (**C**)). Thereafter, seedlings were cultivated in soil for 14 days, followed by phenotypic analysis and photographed, respectively, as shown in (**D**). L7 and L14 of *35S::SiICE1* transgenic *Arabidopsis* and L10 and L17 of *35S::SiICE2* transgenic plants were selected due to relatively higher *SiICE1* or *SiICE2* gene expression in A and B, respectively.

In order to explore the function of *SiICE1* and *SiICE2* in snow lotus, the expression of these two genes was under the control of a CaMV 35S promoter and overexpressed in *35S::SiICE1* and *35S::SiICE2* transgenic *Arabidopsis*. Figure 9 shows the semi-qRT-PCR results of overexpressed *35S::SiICE1* and *35S::SiICE2* transgenic plants (Figure 9A,B). Seven *35S::SiICE1* transgenic plants, L1, L3, L7, L8, L12, L14, and L15, were randomly selected and a specific primer set amplifying *SiICE1* was used to perform semi-qRT-PCR reactions. L14 appeared to express the highest quantity in *35S::SiICE1* plants. In a similar fashion, a semi-qRT-PCR assay was conducted for *SiICE2* expression by using its specific primer set, including L1, L7, L8, L10, L11, L12, L16, and L17 *35S::SiICE2* transgenic plants. Among these, L8 showed the greatest expression. On the contrary, no detectable expression of *SiICE1* and *SiICE2* was obtained in the wild type. The lower panel of Figure 9A,B indicates the relative quantitative expression of semi-qRT-PCR data for *35S::SiICE1* and *35S::SiICE2* transgenic plants, respectively. As shown in Figure 9C,D, similar phenotypes of WT, *35S::SiICE1* (L7, L14), and *35S::SiICE2* (L10, L17) transgenic plants were observed whether these transgenic plants were cultivated in 1/2 MS agar plates (Figure 9C) or in soil (Figure 9D). These data thus revealed that the overexpression of *SiICE1* or *SiICE2* does not affect the seed germination, flowering time, the development of vegetative leaves, inflorescence, the structure of floral organs, or the silique formation (Figure S1).

**Figure 10:**
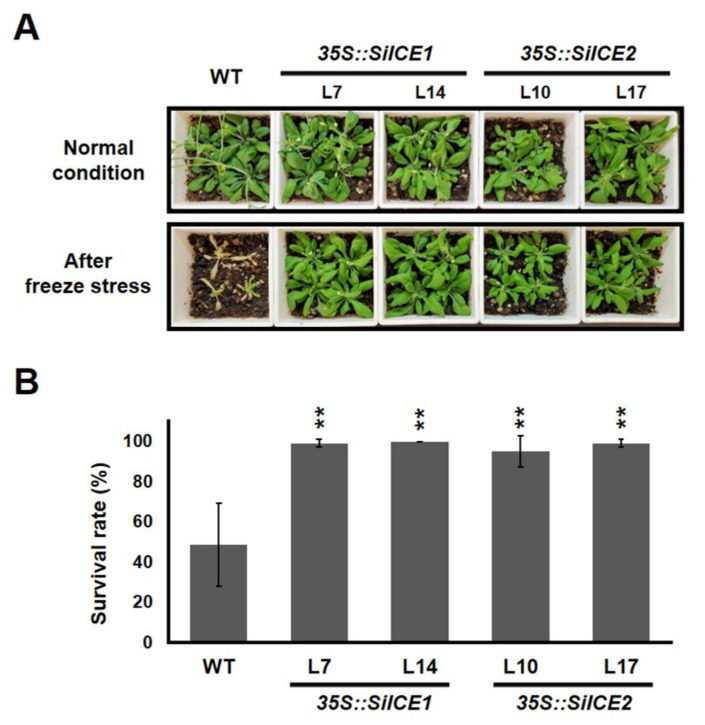
Transgenic *Arabidopsis*, *35S::SiICE1* and *35S::SiICE2*, cultivated in soil and the effect of cold stress on the phenotype and survival rate are displayed. (**A**) Seedlings grown on the 1/2 MS media under 22 °C long-day/light conditions for 14 days were transplanted in soil for further cultivation for 7 days under long-day/light conditions. Thereafter, WT, *35S::SiICE1* (L7, L14), or *35S::SiICE2* (L10, L17) transgenic plants were moved to 0 °C for 24 h, then transferred to 22 °C long-day/light conditions for 7 days in order to recover from the cold/freezing stress. Phenotypes were examined and photographed for both the WT and every transgenic plant. (**B**) Survival rates of the WT, *35S::SiICE1*, and *35S::SiICE2* transgenic plants were calculated after cold/freezing treatment. Six independent experiments were performed and 40 seedlings in each group were analyzed by using the Student’s *t*-test. Asterisks indicate the significant differences in comparison with the control at *p* < 0.01 (**).

Next, to understand the potential capacity of SiICE1 and SiICE2 involved in cold tolerance, WT, *35S::SiICE1* (L7 and L14), and *35S::SiICE2* (L10 and L17) transgenic *Arabidopsis* were first grown in 1/2 MS agar plates. Seedlings grown on the 1/2 MS media under 22 °C long-day/light conditions for 14 days were transplanted into soil for further cultivation for 7 days under long-day/light conditions. Thereafter, WT, *35S::SiICE1* (L7, L14), or *35S::SiICE2* (L10, L17) transgenic plants were moved to 0 °C for 24 h, then transferred to 22 °C long-day/light conditions for another 7 days in order to recover from the cold/freezing stress. The effect of cold/freezing stress (0 °C, 24 h) on the phenotype and the survival rates of the WT and the transgenic plants are exhibited in Figure 10. When compared to those grown at 22 °C, plants cultivated at 0 °C showed a dramatic change in phenotype for the WT, whereas *35S::SiICE1* and *35S::SiICE2* transgenic *Arabidopsis* appeared to possess greater tolerance to cold/freezing treatment and thus showed a similar phenotype to that of the WT (Figure 10A). Survival rates of the WT and each transgenic plant were calculated and shown in Figure 10B. Forty seedlings in each group were analyzed. The survival rate of the WT was 48.61%, while it was 99% and 100% for the *35S::SiICE1* L7 and *35S::SiICE1* L14, respectively. Similarly, the survival rates were 95% and 99% for *35S::SiICE2* L10 and *35S::SiICE2* L17, respectively, indicating that around two-fold higher survival rates were detected in transgenic plants when compared to the WT. As a result, overexpression of *SiICE1* and *SiICE2* increases the cold tolerance in *35S::SiICE1* and *35S::SiICE2* transgenic *Arabidopsis*, suggesting that orthologous *ICE* genes in snow lotus may also function in regulating cold resistance in plants.

### 2.7. Downstream Target Gene Expression Analysis for WT and SiICE Transgenic Arabidopsis

Previous research has revealed that plants with overexpressed *CBF1/DREB1B* and *CBF3/DREB1A* possessed a higher resistance to cold/freezing stress, dehydration, and high-salt treatment [55,56]. In addition, the survival rate of *CBF2* mutants of *Arabidopsis* is higher than that of the wild type (WT) during cold acclimation and cold stress treatment. Interestingly, the rate of dehydration, root elongation, and plant fresh weight of *CBF2* mutants were greater when compared to the WT under drought or high-salt environments, suggesting that CBF2/DREB1C may function as a negative regulator during plant tolerance to cold/freezing, dehydration, or high-salt conditions. Further studies revealed increased *CBF1/DREB1B* and *CBF3/DREB1A* gene expression in *CBF2* mutants, indicating that these two genes are the downstream genes controlled by CBF2. Other studies revealed *LTI78*, *KIN1*, *COR15A*, and *COR47* gene expression was induced earlier and maintained longer at the transcriptional level, while expression of *RCI1A*, *RCI2A*, and *DREB2A* genes related to cold regulation remained unchanged when compared to the WT. The main reason is that no C-repeat (CRT)/dehydration-responsive DNA regulatory element (DRE) cis-elements are located within the promoter region of *RCI1A*, *RCI2A*, and *DREB2A* genes. A complementation assay, produced by transferring an intact *CBF2/DREB1C* gene into *CBF2* mutants, appeared to reduce *CBF1/DREB1B* and *CBF3/DREB1A* gene expression to similar levels as those of the WT. Thus, these data indicate that CBF2/DREB1C functions as a negative regulator which inhibits the expression of *CBF1/DREB1B* and *CBF3/DREB1A* genes, meaning it plays an important role in regulating plant tolerance to cold stress in *Arabidopsis* [57].

**Figure 11:**
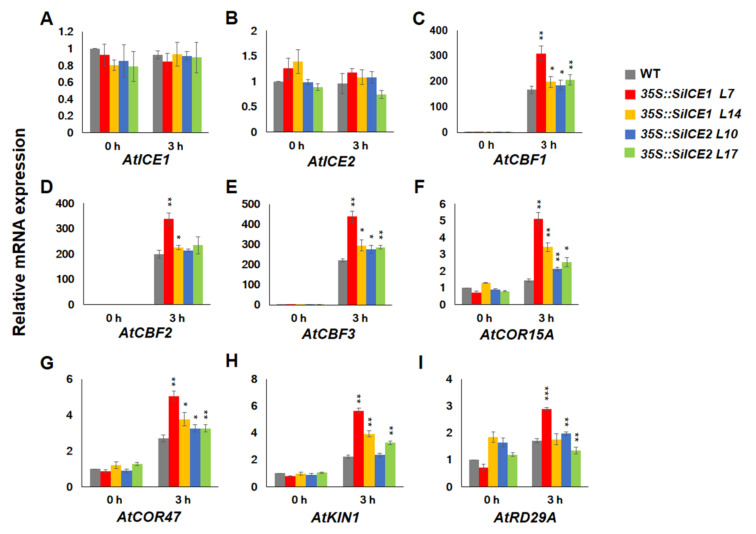
qRT-PCR analysis showing relative mRNA expression of overexpressed *35S::SiICE1* and *35S::SiICE2* transgenic *Arabidopsis* in response to cold/freezing stress. Seedlings, cultivated under long-day/light conditions for 14 days, of WT, *35S::SiICE1* (L7, L14), or *35S::SiICE2* (L10, L17) transgenic plants were moved to −5 °C for 0 h (room temperature control) or 3 h (low-temperature experimental groups), respectively. Gene-specific primers were employed to perform qPCR reactions in order to detect target gene expression at the mRNA level. Target genes included (**A**) *AtICE1*, (**B**) *AtICE2*, (**C**) *AtCBF1*, (**D**) *AtCBF2*, (**E**) *AtCBF3*, (**F**) *AtCOR15A*, (**G**) *AtCOR47*, (**H**) *AtKIN1*, and (**I**) *AtRD29A/COR78*. *Actin2* of *Arabidopsis* was used as a reference control. Each analyzed value is the average of three experimental replicates and the standard deviation (SD) is calculated for each group. Asterisks indicate significant differences at *p* < 0.05 (*), *p* < 0.01 (**), and *p* < 0.001 (***).

In this study, both *35S::SiICE1* and *35S::SiICE2* transgenic plants exhibited greater tolerance to cold stress when compared to the WT (Figure 9 and Figure 10), indicating that some cold-resistance genes may have higher levels of expression during cold treatment. Figure 11 revealed the results of the qPCR analysis for different gene expression levels in the WT (dark-gray bar), *35S::SiICE1* (red and yellow bars), and *35S::SiICE2* (blue and green bars) transgenic plants. Endogenous gene expression of *AtICE1* and *AtICE2* was similar between 0 h treatment (room temperature control) and 3 h cold/freezing stress treatment (Figure 11A,B). On the contrary, the expression of three *CBF* genes, *CBF1*, *CBF2*, and *CBF3*, was found to be significantly increased in response to cold/freezing stress in both *35S::SiICE1* and *35S::SiICE2* transgenic plants by using the qPCR assays (Figure 11C–E). In addition, expression of the downstream target genes of CBF, including *COR15A*, *COR47*, and *KIN1*, was considerably greater in *35S::SiICE1* and *35S::SiICE2* transgenic plants compared to those of the WT during cold/freezing stress for 3 h, but not at room temperature (Figure 11F–H). However, *RD29A/COR78*, a target gene for stress, showed much higher expression in *35S::SiICE1* L7 transgenic plants than those of the WT under cold/freezing stress (Figure 11I (3 h, red bar)). In contrast, others appeared to show no considerable disparity between the WT and the transgenic plants under cold/freezing stress for *RD29A/COR78* gene regulation (Figure 11I (0 h and 3 h)). Our data thus indicate that SiICE1 and SiICE2 may function as positive regulators in *CBF1*, *CBF2,* and *CBF3* gene expression, which in turn contribute to the expression of the downstream target genes *COR15A*, *COR47*, and *KIN1*, thereby leading to greater cold resistance under cold/freezing stress.

## 3. Discussion/Conclusions

Semi qRT-PCR data (Figure 6B) indicate that *SiICE1* and *SiICE2* gene expression was quickly reduced after the temperature was changed from 25 °C to 4 °C (cold stress) for 5 min, although the expression was induced after 10 min cold treatment for snow lotus callus. This sudden reduction in gene expression is possible due to the cold stress causing subsequent oxidative and osmotic stress, which in turn leads to the inhibition of cellular metabolism and activity. The alternative reason could be that cold stress causes the post-translational modification of ICE1, resulting in a subsequent active CBF pathway, but little transcriptional alteration during this process [31,43,58]. Both *SiICE1* and *SiICE2* genes are normally expressed at 25 °C; however, their expression level was lower after cold treatment for 10 min and 30 min for *SiICE1*. On the other hand, the expression level of *SiICE2* was greater after 10 min cold stress, but then decreased after 30 min (Figure 6B). The differential *SiICE1* and *SiICE2* gene expression patterns in response to cold stress suggests that *SiICE1* may function in long-term resistance, while *SiICE2* may be involved in short-term tolerance to a cold environment. Furthermore, it is of interest to note that our results are obtained by using the callus of snow lotus as the experimental material. Whether temporal gene expression of *SiICE1* and *SiICE2* is similar or different in distinct organs of live snow lotus remains unclear.

AtICE1 belongs to the MYC-type bHLH transcription factor family and it recognizes and binds to the MYC cis-element (MYC recognition sites with the consensus sequences of CATNNTG) within the promoter region of a downstream target gene, thereby promoting gene regulation [59]. Five similar MYC cis-elements within the 1 kb promoter region of *CBF3/DREB1A* were predicted by Shinwari’s group [60]. In addition, AtICE1 was identified to be the transcription factor which regulates downstream *CBF3/DREB1A* gene expression [31]. *AtICE2*, on the other hand, is the homologous *ICE* gene which controls downstream *CBF1/DREB1B*, but not *CBF3/DREB1A* gene expression [35]. According to the structural analysis of the full-length amino acids of MYC2 (a MYC-like bHLH transcription factor) and the AtICE1 proteins, a proline-rich domain (PRD), JAZ interaction domain (JID), acidic domain (AD), and the highly conserved acidic region (DDAVDEEVTDTE), which plays an important role in downstream gene activation [61] at the N-terminal end of MYC2, do not exist at the N-end of AtICE1. Instead, ICE proteins contain a serine-rich (S-rich) region at the N-terminus. In order to explore the potential activation domain (AD) of ICE proteins, a yeast one-hybrid assay was performed. Our data (Figure 8) revealed that the potential AD of the AtICE1 and SiICE2 is located at the N-terminal end before the bHLH-ZIP domain. Whether the S-rich region within the N-end is implicated in the target gene activation remains unclear. The AD of the SiICE1 sites is at the bHLH-ZIP domain of this ICE protein. As for the AtICE2 protein, yeast cells grew poorly with full-length, lacking C-terminal ACT, or lacking bHLH/ZIP/ACT domains in the selected agar plates (SD/-Trp-His), suggesting that the AD domain of this ICE protein may not be fully exposed and bind closely to the promoter, resulting in poor His reporter gene expression. Nevertheless, Figure 6 shows that the potential AD domain is located within the N-terminal end of AtICE2.

**Figure 12:**
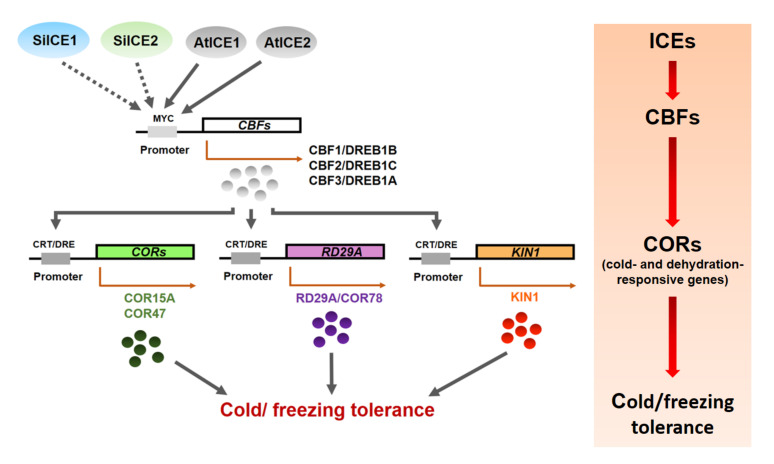
The hypothetical model of SiICE1 and SiICE2 involved in the ICE-CBF-COR transcriptional regulatory network in response to cold/ freezing tolerance. Solid and dotted arrows show the direct and indirect links, respectively. Elliptical objects indicate the functional proteins acting as the transcription factors that control the stress-inducible gene expression. Cis-acting elements in the promoter region that are involved in the stress-responsive transcription are shown in gray boxes. The right panel represents the ICE-CBF-COR controlling pathway in response to cold stress.

Previous studies have revealed that overexpression of *CBF1/DREB1B* and *CBF3/DREB1A* increased plant tolerance to cold/freezing, dehydration, and high-salt stress [55,56]. *CBF2/DREB1C*-related research has shown that *CBF2* mutants survive better than the WT under cold acclimation or cold stress. Similarly, dehydration rate, root elongation, and plant fresh weight were greater in *CBF2* mutants than those of the WT under drought and high-salt stress. These results thus indicate that CBF2/DREB1C behaves negatively in regulating anti-freezing, dehydration, and salt tolerance. Further investigation showed the expression of *CBF1/DREB1B* and *CBF3/DREB1A* indeed increased by a greater degree in *CBF2* mutants than those of the WT. Expression of *LTI78*, *KIN1*, *COR15A*, and *COR47* was induced earlier and longer than those of the WT at the transcriptional level. On the contrary, *RCI1A*, *RCI2A*, and *DREB2A* genes, although related to cold resistance, were not induced in response to the above-mentioned stress. One of the main reasons could be that no C-repeat (CRT)/dehydration-responsive DNA regulatory element (DRE) cis-elements are found in the promoter region of these three genes. In addition, *CBF1/DREB1B* and *CBF3/DREB1A* gene expression behaved similarly to those of the WT once the *CBF2/DREB1C* gene was rescued from *CBF2* mutants by using the complementation assay. Thus, CBF2/DREB1C is a negative regulator which inhibits the expression of *CBF1/DREB1B* and *CBF3/DREB1A*, resulting in subsequent down-regulated cold tolerance in *Arabidopsis* [57]. Our overexpressed SiICE1 and SiICE2 in *35S::SiICE1* and *35S::SiICE2* transgenic plants, respectively, showed a greater tolerance to cold stress when compared to the WT, suggesting some cold resistance genes may be induced during this process. qPCR analysis exhibited that the expression of three *CBF*s, including *CBF1*, *CBF2*, and *CBF3,* was up-regulated in the above-mentioned transgenic plants, whereas expression of the CBF downstream target genes (*COR15A*, *COR47*, *KIN1*, and *RD29A/COR78*) showed slightly different levels. Although *COR15A*, *COR47*, and *KIN1* gene expression was greater at the mRNA level in *35S::SiICE1* transgenic plants, not as much gene expression level was examined when compared to that of the *CBF*s (*CBF1*, *CBF2*, and *CBF3*) in response to cold/freezing stress, as shown in Figure 11. In addition, *RD29A/COR78* is a stress-related target gene whose expression was higher in the transgenic plants than that of the WT at room temperature (Figure 11I (0 h)). However, no consistent up-regulated *RD29A/COR78* gene expression, in contrast to other transgenic *Arabidopsis* with *VaICE1*, *VaICE2*, *ZmmICE1*, *HbICE1*, *ZjICE1,* or *SmICE1* [41,42,43,44,45], was obtained in transgenic plants under cold/freezing stress (Figure 11I (3 h)). Therefore, we propose that *CBF2/DREB1* differentially regulates the gene expression of *CBF1/DREB1B* and *CBF3/DREB1A*, thereby contributing to their different downstream target gene (*COR15A*, *COR47*, *KIN1*, and *RD29A/COR78*) expression. In conclusion, our studies exhibited that SiICE1 and SiICE2 of snow lotus play an important role as positive regulators in cold acclimation. Figure 12 shows the hypothetical pathway of the ICE-CBF-COR controlling pathway in response to cold stress.

## 4. Materials and Methods

### 4.1. Plant Materials and Growth Conditions

Calluses of snow lotus (*Saussurea involucrate*) plants were provided by Dr. Li-Fen Huang, who is a co-author, and cultured as materials for this study. Callus was tissue cultivated at 25 °C under a long-day cycle (16 h light/8 h dark) as instructed by Qiu et al. [48]. Briefly, callus was cultivated for 2 weeks under long-day/light conditions, then subjected to dark treatment for 1 week. These calluses were subsequently divided into two groups. The control group was untreated and cultured at 25 °C, whereas the experimental group was low-temperature treated for 5, 10, or 30 min at 4 °C, respectively.

All *Arabidopsis* plants used in this study were of the Columbia (Col-0) ecotype. *Arabidopsis* ecotype Col-0 was obtained from the Arabidopsis Biological Resource Center (ABRC). *Arabidopsis* plants were grown as described previously [62]. For in vitro growth, seeds were surface-sterilized, sown on 1/2X Murashige and Skoog agar media (MS media) containing 2% (*w/v*) sucrose in Petri dishes, and kept in a growth chamber (22 °C, 16 h light/8 h dark). Seedlings of *Arabidopsis* plants, on the other hand, were grown under a long-day cycle (16 h light/8 h dark) at 22 °C. Seedlings of *Arabidopsis* grown on the 1/2 MS media at 22 °C long day/light for 14 days were then transplanted into soil for further cultivation for another 7 days under long-day/light conditions. Thereafter, WT, *35S::SiICE1* (L7, L14), or *35S::SiICE2* (L10, L17) transgenic plants were moved to 0 °C for 24 h, then transferred to 22 °C long-day/light conditions for 7 days in order to recover from the cold/freezing stress. Phenotypes were examined and photographed for both the WT and every transgenic plant.

### 4.2. Constructing Gene Expression Profile of Snow Lotus Transcriptome to Identify the Potential Gene Candidates Corresponding to Cold Acclimation and Cold Tolerance

According to the published snow lotus transcriptome dataset [48], as well as its original sequencing data downloaded from the Sequence Read Archive (SRA) with the accession number of SRX156202 at the National Center for Biotechnology Information (NCBI) (https://www.ncbi.nlm.nih.gov/sra/SRX156202[accn] (accessed on 6 January 2021)), we employed the CLCbio program to re-construct the gene expression profile of snow lotus transcriptome. Thereafter, the re-constructed gene sequences were used as the searching database in order to investigate the potential gene candidates corresponding to cold acclimation and cold tolerance. SiICE1 and SiICE2 (*Saussurea involucrata* inducer of CBF expression 1 and 2), genes homologous to AtICE1 and AtICE2, respectively, were identified by using this re-constructed transcriptome dataset of snow lotus.

### 4.3. Total RNA Extraction of Snow Lotus Callus and Semi-qRT-PCR

To perform the semi-quantitative RT-PCR assay for the analysis of *SiICE1* and *SiICE2* gene expression, total RNA was isolated from wild-type plants, *35S::SiICE1*, and *35S::SiICE2* transgenic *Arabidopsis*, respectively, by following the manufacturer’s instruction (TRIzol, Invitrogen, Carlsbad, CA, USA). Briefly, 2 μg of extracted total RNA, 1 uL of 50 μM B26T primer (oligo (dT) primer), and 2 μL ddH_2_O were incubated at 65 °C for 5 min, followed by adding 4 uL 5x reaction buffer, 1.5 μL 10 mM dNTP, 1 μL MgCl_2_, 0.5 μL RNasin (Promega, Madison, WI, USA), and 1 μL GoScript^TM^ Reverse Transcriptase (Promega) for reverse transcription (RT) reactions to synthesize cDNA. Subsequently, a polymerase chain reaction (PCR) was conducted by using 1 µL of cDNA and specific primers: forward and reverse primers (Table S1). Amplification conditions were as follows: one cycle at 94 °C for 5 min, then 28 cycles of 94 °C (30 s), 58 °C (45 s), and 72 °C (50 s). The snow lotus *GAPDH* gene was used as the endogenous control. The PCR products were then detected on 1.5% agarose gels. All the primers used in this study are described in Table S1.

### 4.4. Nuclear Localization of Snow Lotus SiICE1, SiICE2, and AtICE1, AtICE2 of Arabidopsis

In order to explore the cellular location of target genes, including *SiICE1*, *SiICE2*, *AtICE1*, and *AtICE2* in the protoplast of *Arabidopsis*, they were constructed with a fluorescent reporter gene (enhanced GFP, eGFP) at the N-terminal end in an expression vector, pK7WGF2, provided by Dr. Choun-Sea Lin (Agricultural Biotechnology Research Center, Academia Sinica, Taipei, Taiwan). Full-length cDNAs of these target genes were then constitutively expressed, resulting in GFP-SiICE1, GFP-SiICE2, GFP-AtICE1, and GFP-AtICE2 fusion proteins expressed under the control of the CaMV35S promoter. A AtCO-mCherry recombinant fusion plasmid carrying a *CONSTANS* (*AtCO*) gene and a fluorescent mCherry reporter gene was used as a nucleus marker. These reporter constructs were isolated and transformed into protoplast cells using the Tape-*Arabidopsis* Sandwich method [63]. Fluorescence in the transformed cells was observed on an Olympus Fluoview FV1000 (Olympus, Tokyo, Japan) confocal microscope and the images were photographed and recorded by a NIS-Elements Viewer. The locational relationship between the nuclear control AtCO and the different ICE proteins was examined.

### 4.5. Yeast One-Hybrid Analysis and Spotting Assay

The full-length cDNA for all the above-mentioned target genes, including *SiICE1*, *SiICE2*, *AtICE1*, and *AtICE2*, were each generated by PCR using the gene-specific primer sets. The PCR fragments were then ligated into the plasmid pGBKT7 (GAL4 DNA-binding domain vector, GAL4DB vector). Subsequently, yeast one-hybrid analyses were carried out according to the instructions of Frozen-EZ Yeast Transformation II^TM^ (ZYMO Research, Orange, CA, USA). Specific constructs were then transformed into the yeast strain AH109 which contains a *Histidine* reporter gene (*HIS3*). DNA (upstream activating sequences (UASs) and TATA boxes of the promoter region of a *HIS3* reporter gene) and protein (GAL4DB target gene fusion protein) interactions were determined by the growth conditions on the selective synthetic defined medium lacking tryptophan and histidine (SD/-Trp-His) as well as on the control (SD/-Trp) medium. DNA–protein interactions were finally determined by the spotting assays. All the spotting assays were repeated at least three times.

### 4.6. Overexpression of Recombinant Plasmid pEpyon-3bk Carrying SiICE1 and SiICE2

Plasmid vector pEpyon-3bk was obtained from Dr. Chang-Hsien Yang (Graduate Institute of Biotechnology, National Chung Hsing University, Taichung, Taiwan). This is a binary vector whose transformation is carried out by *Agrobacterium tumefaciens*, and whose target gene expression is under the control of a CaMV 35S promoter, leading to overexpression of the target gene at the transcriptional level. Moreover, a VP16 (PPTDVSLGDEL) segment, functioning in the enhanced transcription, was added to the 3′ end of the target genes, resulting in pEpyon-SiICE1 and pEpyon-SiICE2 recombinant plasmids, respectively. These recombinant DNAs were then separately transformed into competent *Agrobacterium* cells, followed by infecting *Arabidopsis* with these transformed *Agrobacterium* by using the floral-dipping method [64]. Thus, *SiICE1* and *SiICE2* transgenic *Arabidopsis* are expected to constitutively overexpress, respectively, SiICE1 and SiICE2, and their potential biological function is subsequently examined by phenotypic analysis, including seed germination, size of the seedlings, leaf development, flowering time, structure of the inflorescence and floral organs, fruit growth, and seed formation. All was photographed and recorded in detail, as shown in Figure S1.

### 4.7. Real-Time Quantitative RT-PCR (qRT-PCR)

Expression analysis of cold-responsive genes in transgenic plants that were exposed to the cold condition described above and total RNA (2 μg), extracted from the leaves of wild-type and *35S::SiICEs* transgenic *Arabidopsis* plants, was used for cDNA synthesis by reverse transcription of a 15 μl reaction mixture using the GoScript^TM^ Reverse Transcriptase (Promega, Madison, WI, USA) according to the manufacturer’s protocols. Quantitative RT-PCR (qRT-PCR) was performed with gene-specific primers, including *AtICE1*, *AtICE2*, *AtCBF1*, *AtCBF2*, *AtCBF3*, *AtCOR15A*, *AtCOR47*, *AtKIN1*, and *AtRD29A/COR78*. *AtActin2* was used as an internal control. One microliter of the cDNA sample (10 × dilution) from the above RT reactions was further used for the qPCR reaction as follows: 94 °C for 3 min, followed by 40 cycles of 94 °C for 20 s, 60 °C for 30 s, and 72 °C for 15 s. The qRT-PCR was performed on a Roche LightCycler^®^-480 real-time PCR system by using the KAPA SYBR FAST Universal qPCR Kit (KAPA BIOSYSTEMS) as instructed by the manual’s recommendations. The comparative 2^−^^ΔΔCt^ method was finally employed to determine the relative gene expression level. The value for the untreated wild-type was normalized to 1. Each biological sample was performed with three technical repetitions, and data analyses were carried out using three independent biological replicates. All primers used in this study are listed in Table S1. Values were statistically analyzed by ANOVA or the Student’s t-test to calculate the statistical significance.

## Data Availability

Not applicable.

## Supplementary Materials

The following are available online at https://www.mdpi.com/article/10.3390/ijms221910850/s1. Table S1. Primers used in this study. Figure S1. Phenotypic analysis of 35S::SiICE1 (L7 an L14) and 35S::SiICE2 (L10 an L17) transgenic Arabidopsis. (A) Phenotype of 4-day-old plants were germinated and grown in 1/2 MS agar plates. Bar = 1 cm. (B) Percentage of plants that enters the flowering stage (flowering time) is quite similar for these transgenic Arabidopsis. (C) Phenotypes of the inflorescence structure of different seedlings which were cultivated in soil for 3 weeks after transferred from the 1/2 MS agar plates. Bar = 1 cm. (D, E, F) Phenotypes of floral organs, siliques and seeds, respectively. No obvious difference in morphology, shape and color between WT and the transgenic Arabidopsis was observed. Bar = 0.5 mm shown in (D). Bar = 2 mm shown in (E). Bar = 1 mm shown in (F).
