# Supplementary material for: Saussurea involucrata (Snow Lotus) ICE1 and ICE2 Orthologues Involved in Regulating Cold Stress Tolerance in Transgenic Arabidopsis
Source: Int J Mol Sci. 2021 Oct 7;22(19):10850. doi: 10.3390/ijms221910850 (PMC8509503; doi:10.3390/ijms221910850)
Supplement: Supplementary file 1 [file ijms-22-10850-s001.zip › ijms-1376961-supplementary.pdf]

International Journal of *Molecular Sciences*

MDPI

Supplementary Materials: *Saussurea involucrata* (Snowlotus) *ICE1* and *ICE2* orthologues involved in regulating cold stress tolerance in transgenic *Arabidopsis*

Chia-Ling Wu[1,+], Lee-Fong Lin[1,+], Hsiao-Chun Hsu[2], Li-Fen Huang[3], Chung-Der Hsiao[4] and Ming-Lun Chou [1,*]

Table S1. Primers used in this study.

Gene	Forward primer (5' to 3')	Reverse primer (5' to 3')	Purpose
AtICE1	ATGGGTCTTGACGGAAACAATGGTGGAGGGGT	TCAGATCATACCAGCATACCCTGCTGTATCGA	RT-PCR
AtICE2	ATGAACAGCGACGGTGTTTGGCTTGAC	TCAAACCAAACCAGCGTAACCTGCTGT	RT-PCR
SiICE1	ATGTTATCGAGAGTTAACAACAACACT	TCAAGCGACACCATGGTAACCCGCAGA	RT-PCR
SiICE2	ATGTTATCAGAAGACAACATTGGTGTTTGGAT	CTAAGCCACACCATGGTAGCCGGCCGATTCCA	RT-PCR
SiGAPDH	TAGCAAGGATGCTCCCATGTTCGT	TCACATAAAGTCTGTGGATACAACATC	RT-PCR
AtActin	GGCTAACAGAGAGAAGATGACC	AATAGACCCTCCAATCCAGAC	RT-PCR
AtICE1-FL	AAAAAGCAGGCTTCATGGGTCTTGACGGA	AGAAAGCTGGGTCTCAGATCATACCAGC	Gateway cloning
AtICE1/-ACT	AAAAAGCAGGCTTCATGGGTCTTGACGGA	AGAAAGCTGGGTCTCAGTTCACTGCTCTTCC	Gateway cloning
AtICE1/-HLH/ ZIP/ACT	AAAAAGCAGGCTTCATGGGTCTTGACGGA	AGAAAGCTGGGTCTCACATACCTTTCTTCTT	Gateway cloning
AtICE2-FL	AAAAAGCAGGCTTCATGAACAGCGACGGT	AGAAAGCTGGGTCTCAAACCAAACCAGC	Gateway cloning
AtICE2/-ACT	AAAAAGCAGGCTTCATGAACAGCGACGGT	AGAAAGCTGGGTCTCAGTTCACTGCCTTTCC	Gateway cloning
AtICE2/-HLH/ ZIP/ACT	AAAAAGCAGGCTTCATGAACAGCGACGGT	AGAAAGCTGGGTCTCACATTCCTTTCTTCTT	Gateway cloning
SiICE1-FL	AAAAAGCAGGCTTCATGTTATCGAGAGTT	AGAAAGCTGGGTCTCAAGCGACACCATG	Gateway cloning
SiICE1/-ACT	AAAAAGCAGGCTTCATGTTATCGAGAGTT	AGAAAGCTGGGTCTCAATTAACTGCTCTTCC	Gateway cloning
SiICE1/-HLH/ ZIP/ACT	AAAAAGCAGGCTTCATGTTATCGAGAGTT	AGAAAGCTGGGTCTCACAGCCCTTTTTTCTT	Gateway cloning
SiICE2-FL	AAAAAGCAGGCTTCATGTTATCAGAAGAC	AGAAAGCTGGGTCCTAAGCCACACCATG	Gateway cloning
SiICE2/-ACT	AAAAAGCAGGCTTCATGTTATCAGAAGAC	AGAAAGCTGGGTCCTAATTCACCGCTTTTCC	Gateway cloning
SiICE2/-HLH/ ZIP/ACT	AAAAAGCAGGCTTCATGTTATCAGAAGAC	AGAAAGCTGGGTCCTACAACCCTTTCTTCTT	Gateway cloning
SiICE1	GCTCTAGAATGTTATCGAGAGTTAACAACA	GGGGTACCAGCGACACCATGGTAACCCGC	XbaI/KpnI cloning
SiICE2	GCTCTAGAATGTTATCAGAAGACAACATTG	GCTCTAGAAGCCACACCATGGTAGCCGGCC	XbaI cloning

AtICE1-qRT	CTTCCATCCGTTGACACCTAC	CTCTAGCTTGCTGGCCTTTAG	Real-time qPCR
AtICE2-qRT	TCCACAAACGCTGTCTTACC	GTTCACTGCCTTTCCTTCTCT	Real-time qPCR
AtCBF1-qRT	GAGACGATGGTGGAAGCTATTT	AGCATGCCTTCAGCCATATTA	Real-time qPCR
AtCBF2-qRT	GACCTTGGTGGAGGCTATTT	ATCCCTTCGGCCATGTTATC	Real-time qPCR
AtCBF3-qRT	GACGTTGGTGGAGGCTATTT	AGCATCCCTTCTGCCATATTAG	Real-time qPCR
AtCOR15A-qRT	GGCGTATGTGGAGGAGAAAG	CCCTACTTTGTGGCATCCTTAG	Real-time qPCR
AtCOR47-qRT	GGCTGAGGAGTACAAGAACAA	ACAATCCACGATCCGTAACC	Real-time qPCR
AtKIN1-qRT	GCAATGTTCTGCTGGACAAG	TCCTTCACGAAGTTAACACCTC	Real-time qPCR
AtRD29A-qRT	GCTTTCTGGAACAGAGGATGTA	CGACTCTTCCTCCAACGTTATC	Real-time qPCR
AtActin-qRT	GGTAACATTGTGCTCAGTGGTGG	AACGACCTTAATCTTCATGCTGC	Real-time qPCR

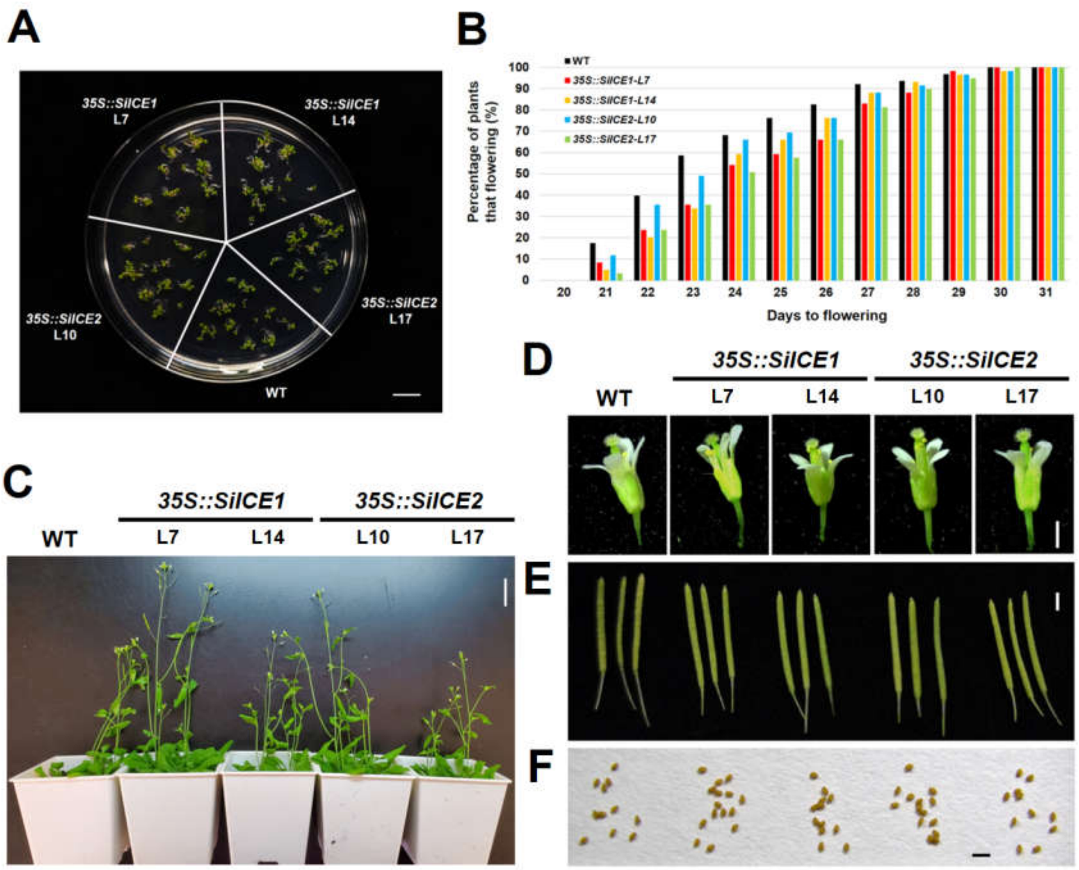

Figure S1. Phenotypic analysis of *35S::SiICE1* (L7 an L14) and *35S::SiICE2* (L10 an L17) transgenic *Arabidopsis*. (**A**) Phenotype of 4-day-old plants were germinated and grown in 1/2 MS agar plates. Bar = 1 cm. (**B**) Percentage of plants that enters the flowering stage (flowering time) is quite similar for these transgenic *Arabidopsis*. (**C**) Phenotypes of the inflorescence structure of different seedlings which were cultivated in soil for 3 weeks after transferred from the 1/2 MS agar plates. Bar = 1 cm. (**D, E, F**) Phenotypes of floral organs, siliques and seeds, respectively. No obvious difference in morphology, shape and color between WT and the transgenic *Arabidopsis* was observed. Bar = 0.5 mm shown in (D). Bar = 2 mm shown in (E). Bar = 1 mm shown in (F).
